# Graduate Study on the Continent—Austria

**Published:** 1908-10-31

**Authors:** 


### GRADUATE STUDY ON THE CONTINENT—AUSTRIA.

### II.—VIENNA.

THERE is perhaps no European city that, among English-speaking students at least, enjoys as great a fame as a centre for graduate study in medicine as Vienna. Whereas Paris and Rome, Milan and Berne number their ones or twos of English graduate students, and Munich and Berlin their dozens, the Austrian capital can proudly point to scores. This is no mere statement founded on the passing impressions of a visit; it is a fact demonstrable by reference to the University statistics. When we take up the last report of the Vienna University we find that during the winter term 1907-1908 there were 1,492 matriculated medical students and 506 guest students attending the lectures. Of the former only three were English-speaking, but of the latter 113 were Americans and five English. When we look down the list of "extraordinary students" (guest students) we are at once struck by this preponderance of English-speaking students. It is not as at Berlin, where the various foreign nationalities among the post-graduate students show no great preponderance of any one (with the exception, perhaps, of Russians). At Vienna the American students are far in the majority. To their 113, Russia only sends 34, and no other country reaches double figures. Among English students the preference for Vienna is equally marked, though of late years such centres as Munich and Berlin have begun to rival it in popularity. But the English graduate still thinks it a mark of special fame to be able to put Vienna in brackets after his name in the directory.*

That Vienna attracts so many English-speaking students is due to various causes. To begin with, it was one of the first centres which gave facilities for post-graduate study. It possessed a brilliant group of teachers, whose names were famous in all departments of medical science. "The Vienna School" is historical. It led; it did not follow. Its great men gathered round them brilliant nebulæ of lesser stars, who carried their fame literally to the ends of the civilised world. Just as at one time, before the German schools reached their present fame, the Universities of Holland and France attracted students, so Vienna attracted pupils from far and near during the last century. One centre rises and the other falls: the popularity of one means the decline of another; but decline and decay do not seem to have materially affected the reputation of Vienna. Thus at the time when the late Professor Billroth was undoubtedly the foremost surgeon in Europe, Vienna was unquestionably the chief centre for surgical study. But at Billroth's death matters changed. Mikulicz at Breslau, Bergmann at Berlin, Kocher at Berne, and Réclus at Paris divided the honours between them, and no one can honestly claim that Vienna to-day possesses the same reputation as a school of surgery as it possessed during the lifetime of Billroth. It still, however, lives on its former reputation, just as some of our London medical schools do. The graduate who knew Vienna in its prime and studied there is still its staunch supporter. For him there is no other place like it. "You must go to Vienna," he says to his young graduate friend, forgetting that times are not what they used to be in his day, and that other centres have arisen which claim an equal consideration and equal respect.

Then, again, students go to Vienna because they do not know the attractions of other places. It is the best-advertised centre because its graduate students are always advertising it. What, then, are the claims of Vienna? It is a large centre: of that there can be no doubt whatever. It possesses seventeen clinics and large hospitals which are open to the graduate student, and even these, large and commodious as they are, cannot adequately deal with the enormous amount of clinical material. There are many places of clinical and general interest: the institutions are large and fine, and there is every opportunity for research work. It is still a leading centre in the medical world. Above all, it offers the student a prodigious number of ordinary and special courses, and it gives him facilities for practical work such as are certainly not to be found in England. On the other side of the picture Vienna is one of the most expensive centres which the student can choose, and every year its expensiveness is rising. Living

* We may here remark that we consider such references to foreign schools as for the most part misleading. No one has the right to put his graduate centre on an equal footing with his *alma mater*, and it would be well if the publishers of the Medical Directory limited such bracketed "places of education" to one reference only.

is far dearer than in Berlin or Paris, while additional expenses are much heavier than in the German centres. It is true that the average course is slightly cheaper than at Berlin, but in the long run even that advantage is counterbalanced by the other items on the student's balance-sheet. Rooms vary from 40 to 60 kronen per month, without extras; *pensions*, of which there are very many, average £8 per month, also without extras. The student will find that he cannot very well get along, even for a prolonged stay, unless he allows himself double of what he would allow himself in Berlin, or a third more than what he would allow himself in London for living expenses. In no other city is the tipping system so widely developed as here, and the new comer soon finds out that there is a host of extras for which he has not bargained. Thus he cannot obtain a latch-key. Whenever he comes home after ten o'clock at night he has to ring up the porter, whose scheduled tip for opening the door is twopence. At comparatively few of the restaurants can he obtain table d'hôte; he must always dine à la carte. Small expenses such as tram fares (it is customary to tip the conductor), boot-cleaning, lighting, and tips to servants, porters, and attendants, rapidly swell his account. We lay stress on these points because it is still frequently stated that study at Vienna is very cheap. It is nothing of the kind, for the post-graduate at least. In the matter of outlay Berlin is far superior to Vienna, and the other Austrian centres even more so. For the student who need have no financial considerations to debar him from enjoying to the full the attractions here, it is, of course, a delightful place, worthy to rank with Paris as a city for pure enjoyment of life, but for the average graduate student, who desires to get the maximum benefit from the minimum outlay, it is scarcely a centre to be recommended.

